# Retreating marsh shoreline creates hotspots of high-marsh plant diversity

**DOI:** 10.1038/s41598-019-42119-8

**Published:** 2019-04-08

**Authors:** Tracy Elsey-Quirk, Giulio Mariotti, Kendall Valentine, Kirk Raper

**Affiliations:** 1 Department of Oceanography and Coastal Sciences, Louisiana State University, Baton Rouge, LA USA; 2 Academy of Natural Sciences of Drexel University, Department of Biodiversity, Earth and Environmental Science, 1900 Benjamin Franklin Parkway, Philadelphia, PA 19103 USA; 3 Center for Computation and Technology, Louisiana State University, Baton Rouge, LA USA

## Abstract

Marsh edge retreat by wave erosion, an ubiquitous process along estuaries, could affect vegetation dynamics in ways that differ from well-established elevation-driven interactions. Along the marshes of Delaware Bay (USA) we show that species composition from marsh edge to interior is driven by gradients in wave stress, bed elevation, and sediment deposition. At the marsh edge, large wave stress allows only short-statured species. Approximately 17m landward, decreasing wave stress and increasing deposition cause the formation of a ridge. There, high marsh fugitive and shrub species prevails. Both the marsh edge and the ridge retreat synchronously by several meters per year causing wave energy and deposition to change rapidly. Yet, the whole ecogeomorphologic profile translates landward in a dynamic equilibrium, where the low marsh replaces the high marsh ridge community and the high marsh ridge community replaces the mid-marsh grasses on the marsh plain. A plant competition model shows that the disturbances associated with sediment deposition are necessary for the high marsh species to outcompete the mid-marsh grasses during rapid transgression. Marsh retreat creates a moving framework of physical gradients and disturbances that promote the co-existence of over ten different species adjacent to the marsh edge in an otherwise species-poor landscape.

## Introduction

Coastal marshes are a transitional ecosystem between the land and the sea, providing a number of important ecological services including habitat for ecologically and economically important species, sequestration of carbon and other pollutants, and coastal protection^1^. In a regime of rising sea-level and anthropogenic modifications, however, marshes are increasingly experiencing deterioration and loss. Even in the absence of sea-level rise, mature salt marshes, which tend to slope steeply to the intertidal flat, are being lost due to inherent lateral retreat by wave-induced edge erosion^2,3^. As marshes retreat laterally, low marsh plant species are predicted to migrate landward at the expense of mid- and high marsh species, particularly in areas with barriers to inland migration^4,5^. Loss of high marsh species at the upland boundary can significantly lower overall vegetation diversity and complexity, as low elevations tend to be dominated by one or a few stress-tolerant species.

Coastal wetlands are ideal systems to test hypotheses of drivers of plant community patterns due to strong gradients in abiotic conditions, relatively low species richness, and striking plant zonation^6–8^. Plant community dynamics in salt marshes have been determined primarily for the relatively stable marsh interior, where discrete disturbances are infrequent. In the marsh interior, competitive interactions tend to increase as tidal flooding and salinity decline, generally regulated by elevation^8–10^. Less competitive, stress-tolerant species, such as *Spartina alterniflora*, are relegated to frequently-flooded low marsh elevations by competitive exclusion by mid-marsh species such as *Spartina patens*^8,10^. Similarly, high marsh shrubs are limited by the physical stresses at lower elevations and outcompete marsh grasses in areas of reduced flooding^11,12^. Therefore, sea-level rise and increased inundation generally causes the replacement of mid- and high marsh species with low marsh grasses^13,14^.

An exception to the paradigm of elevation-driven competition might be present at the marsh-estuary boundary, where physical processes due to waves create frequent disturbances that may affect plant community structure and species interactions^15^. Waves directly shear the vegetation and rework the soil surface, an effect that is greatest at the marsh edge and declines exponentially inland^16^. Waves also have an indirect effect of depositing sediments and plant debris (i.e., wrack), which can smother existing plants^17,18^ and provide opportunities for rapid colonization by fugitive and high marsh species ^sensu^^8,19^. Along these high-energy marsh shorelines, frequent wave-induced disturbances along with gradients in elevation may control vegetation patterns, whereby community structure and species diversity depend on the frequency and intensity of disturbances, species-specific growth rates, and niche preferences^20^. These interactions, however, have yet to be described or modeled for high-energy marsh edge environments.

At the estuary-marsh transition, physical disturbances are also compounded by a retreating marsh edge boundary, which forces the vegetation to rapidly adapt to changing physical conditions. This setting contrasts with the channel-interior marsh boundary, which remains relatively stable over time and only varies gradually with sea-level rise. Given that rates of marsh edge retreat can be up to 10 meters per year^21,22^^present study^ (Table S1), it is unclear whether the vegetation near the marsh edge is simply lost in succession or adapts. With the inherent retreat of marsh boundaries when sediment input is less than what is eroded^2,3^, characterizing linkages between vegetation patterns and underlying morphodynamics is essential for a mechanistic understanding of succession and species movement patterns in rapidly changing estuary-marsh landscapes.

With this study, we aimed to examine how intense physical drivers interact with biotic interactions to influence vegetation structure (i.e., species composition, richness, height, and density) along a rapidly retreating the marsh-estuary boundary. A simple plant competition model was developed to explain field observations that included both physical disturbances and interactions among species. Previous models have simulated competition between multiple salt marsh plant species^23,24^, but have not accounted for physical disturbances other than those associated with inundation. In addition, previous plant competition models^23–25^ have assumed that the parameters driving the stochastic vegetation dynamics adapt instantaneously to the relative fitness, an assumption that might not hold where ecotones are migrating at extremely fast rates (e.g., >10 m yr^−1^).

## Dynamics of The Marsh Edge and Marsh Ridge

**Figure 1:**
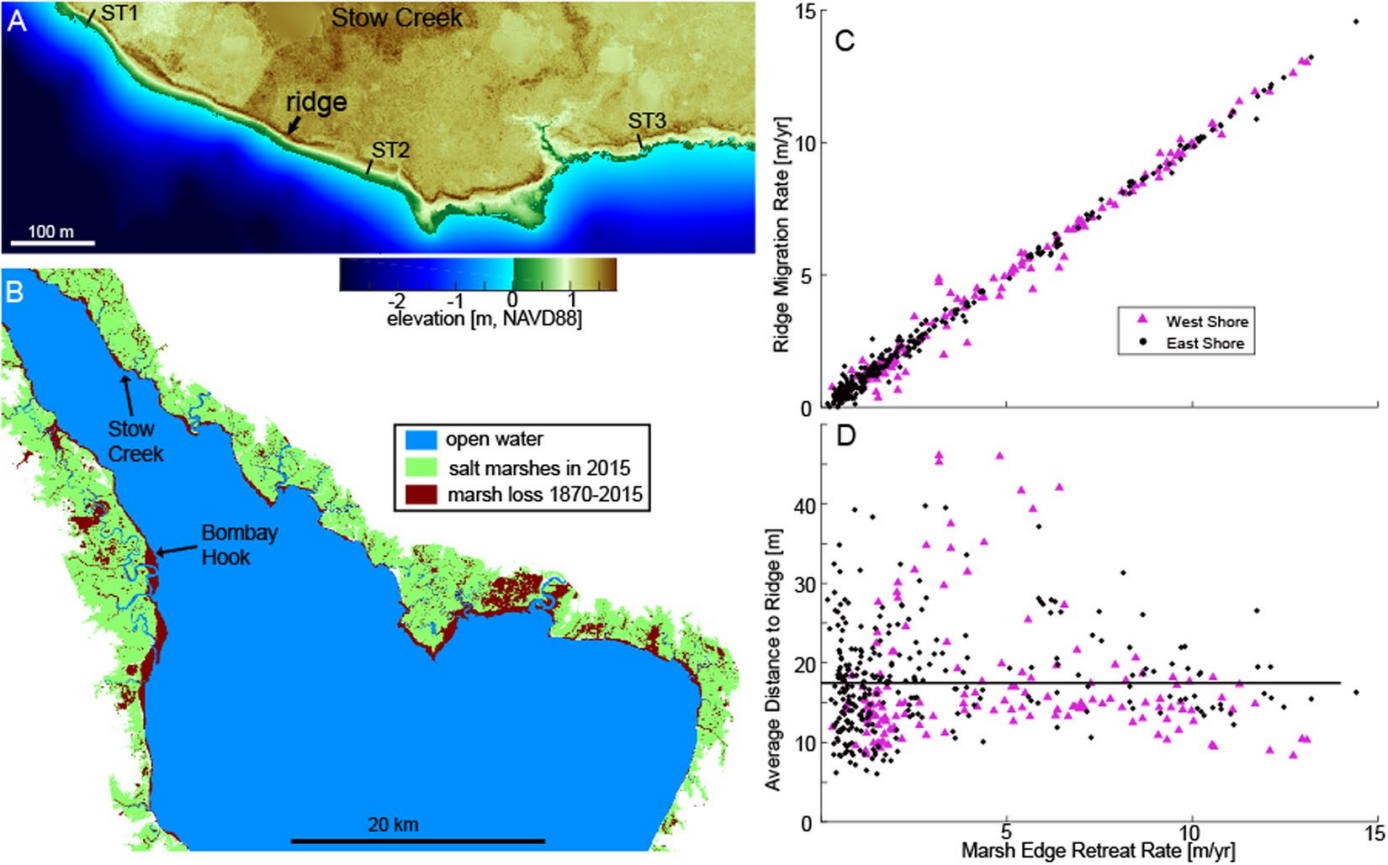
(**A**) LIDAR elevation map of Delaware Estuary showing the sediment ridge and three of the ten field transects; (**B**) Study sites and marsh loss from ~1870 to 2015 (marsh loss map with 30 m resolution was generated using arcGIS 2.8 and Matlab R2015b, with raw data from NOAA T-sheets historic surveys and NASA Landsat); (**C**) Relationship between marsh edge erosion rate and ridge migration rate (1991–2017); and (**D**) Average distance from the estuary-marsh boundary to sediment ridge as a function of marsh edge retreat rate. The line indicates the average distance to the ridge across the estuary.

**Figure 2:**
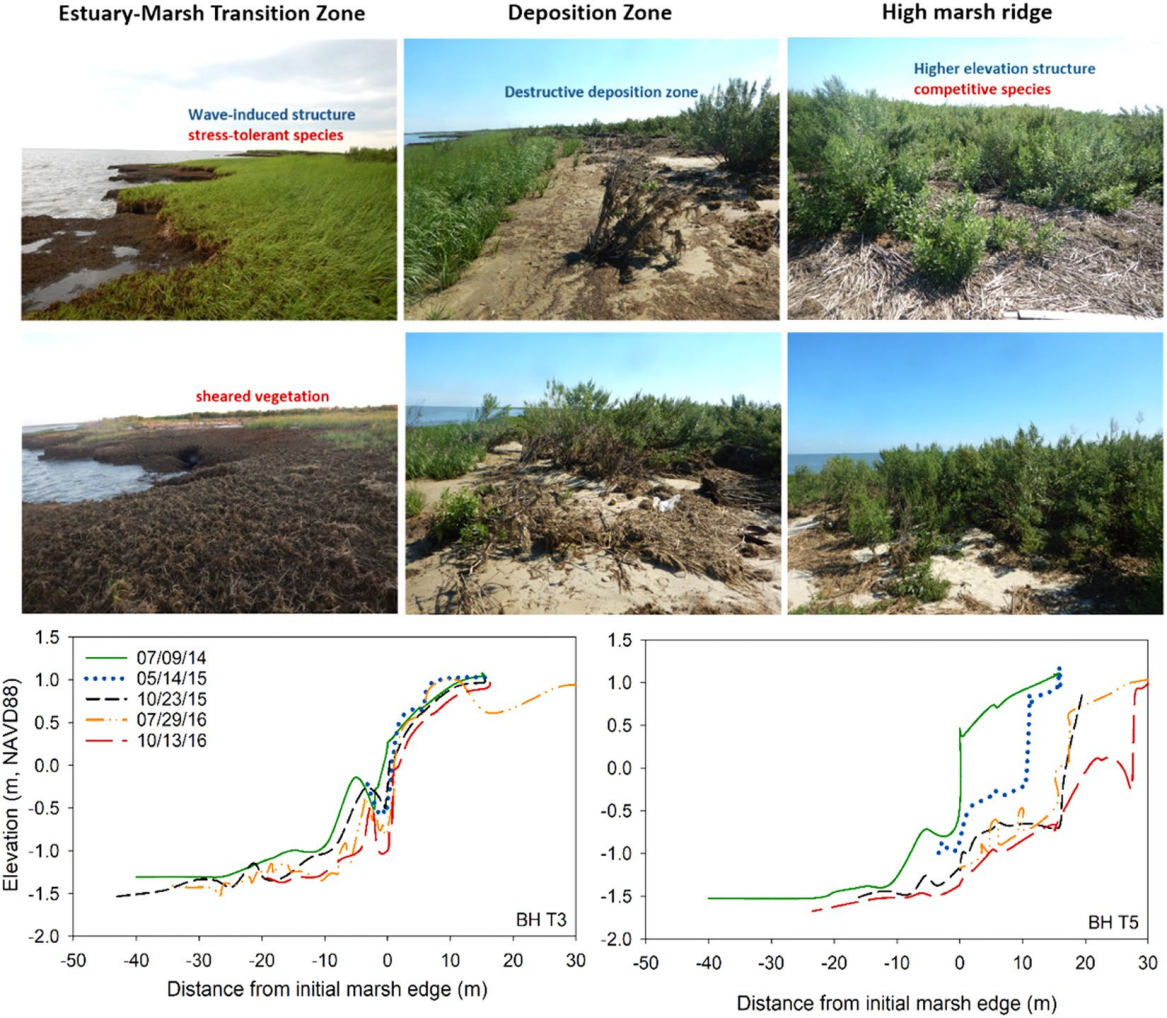
Images representing gradients in physical disturbances (i.e., waves and deposition) from the estuary–marsh boundary to the high marsh ridge (top). At the estuary-marsh transition zone, the vegetation consists of stress-tolerant species, *S. alterniflora*, or is sheared by wave energy. Averaging 16 m inland, the Deposition Zone contains deposits dominated by fine sands and secondarily plant fragments (i.e., wrack), which can smother existing vegetation and allow the landward encroachment of *S. alterniflora*. Immediately landward, competitive high marsh fugitive species occur on the sediment ridge. Examples of marsh edge profile retreat based on repeated RTK surveys (bottom). Photo credit: T. Quirk.

The Delaware Estuary is a mesotidal drowned valley undergoing marine transgression as a result of relative sea level rise, which in the last century has averaged 3.5 mm y^−1^ ^26^. The fringing marshes bordering the estuary are characterized by a steep scarp that – subject to energetic swell and sea waves – has been retreating at a rate of 1.2 ± 0.1 m y^−1^ over the long-term (1879–2012) and 2.8 ± 0.5 m y^−1^ more recently (2007–2012)^27^. Marsh edge erosion, which is common in marshes around the world, has here created a prominent feature: a 0.47 ± 0.02 m high and 3–10 m wide wash-over ridge bordering much of the wetland-dominated lower estuary (Figs 1 and 2).

**Figure 3:**
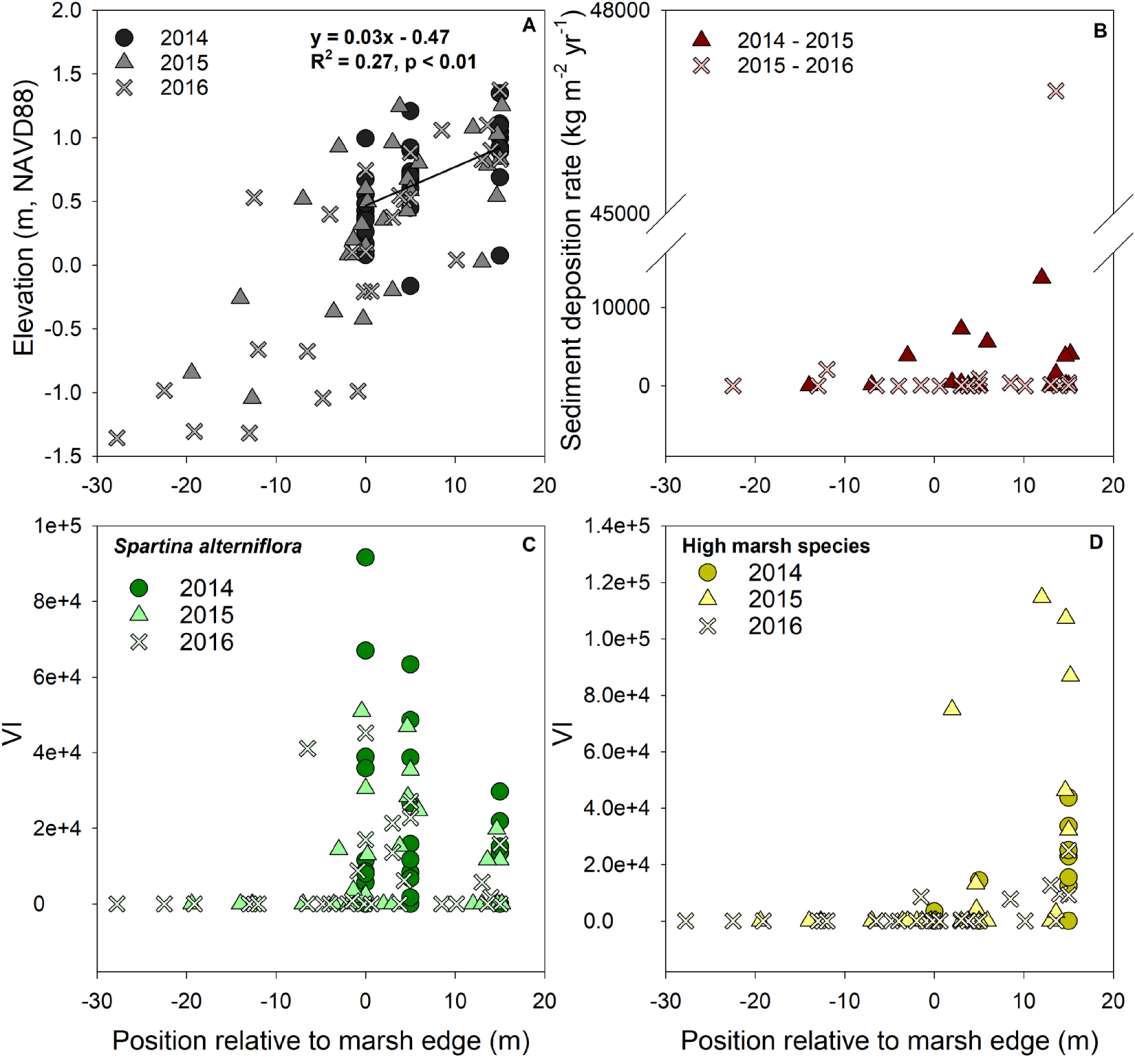
Marsh elevation (**A**), sediment deposition rate (**B**), and summertime vegetation structure index (VI) of the low marsh stress-tolerant species, *S. alterniflora* (**C**) and the high marsh plant community (**D**) at the estuary-marsh transition over a three year period.

Field observations indicate that the ridge is composed primarily of fine sand resuspended from the tidal flat and secondarily of stem fragments of *Phragmites australis* (i.e., wrack); rarely did any muds of marsh origin accumulate. Bay-wide estimates from 2017 indicate that the sediment ridge averaged 17.5 ± 0.3 m from the marsh edge (Fig. 1D). The average occurrence of the ridge at approximately 17 m is supported by field data, where only small quantities of sediment accumulated at plots originating 5 m from the marsh edge (48 ± 36 kg m^−2^ yr^−1^), while sediment deposition was an order of magnitude greater at 15 m (464 ± 234 kg m^−2^ yr^−1^; Fig. S1). This location was characterized by large episodic deposition events including 8,058 kg m^−2^ of wrack and sediment (60% organic matter by weight) between March and May 2015 and 47,580 kg m^−2^ of fine sand between May and August 2016, the latter equivalent to over 1 m of sediment accretion (Figs 3 and S2). Similarly, surface accretion rate was over 7 times greater at a 15 m distance (7.6 ± 3.5 cm yr^−1^) than 5 m from the marsh edge (<1 cm yr^−1^; Fig. S1).

At the estuary-scale, the ridge migrated landward at a rate close to the rate of marsh edge retreat over a range of 0 to 15 m yr^−1^ (Fig. 1C), indicating near instantaneous adaptation of the ridge to wave erosion and sediment deposition. Although the distance between the marsh edge and the ridge remained nearly constant at each location over time, the distance varied spatially within the estuary from 10 to about 40 m (Fig. 1D). The distance of the ridge from the marsh edge was not correlated with the rate of marsh edge retreat or with the orientation of the coastline (Fig. 1D).

### Plant community patterns

Vegetation within approximately 10 m of the marsh edge was either sheared or comprised of short-statured stress-tolerant, *S. alterniflora* (Figs 2 and 3C). The occurrence of *S. alterniflora* close to the marsh edge demonstrates the ability of this species to withstand not only frequent flooding at low elevations, but also the direct physical stresses imposed by waves. At approximately 15 m distances, an abrupt change in vegetation is represented by a reduction in *S. alterniflora*, and the novel occurrence of high marsh and fugitive species including *Atriplex patula*, *Bolboschoenus robustus, Iva frutenscens*, *P. australis*, *Solidago sempervirens*, and *Spartina cynosuroides* (Figs 2, 3; Figs S1, S3 and S4) – plant species that are typically found seaward of the terrestrial border or along levees or drainage ditches. *Iva frutescens*, for example, is found at elevations of 1.5 m above mean sea level or where water tables average greater than 10 cm depth^11^. Similarly, *P. australis* is limited to colonizing high marsh habitats with low soil salinities and high soil oxygen levels^28^. In the Delaware Estuary, high marsh species other than those present on the ridge are generally located in the high marsh/upland boundary more than several km away from the marsh edge.

In order to concisely quantify the vegetation pattern, we calculated the Vegetation Structure Index (VI) as the product of species richness, mean canopy height, and stem density ^after^^29^. This parameter gives intentional weight to changes in the number of species, and similarly reflects the dynamics of each structural component (Fig. S5). Abrupt changes in vegetation structure (VI) and species composition coincided with wave-driven changes in topography and sediment deposition over time (Fig. 3). *Spartina alterniflora* was more frequent, taller, and denser closer to the marsh-estuary boundary, where elevations and deposition rates were lower (Fig. 3). High marsh species had high VI around 15 m from the edge, where elevation and deposition rates were higher (Fig. 3).

High elevations created by sediment deposits are also accompanied by a disturbance effect where deposition eliminates competition and creates an open niche allowing the opportunistic colonization of high marsh perennials, annuals, and fugitive species. Ridge transgression, therefore, initiates a sequence of ecological succession with the colonization of *S. alterniflora* at the abandoned seaward edge where high marsh species are lost due to wave exposure, smothering of mid-marsh grasses on the landward edge of the ridge, and colonization and persistence of high marsh species on the ridge.

Vegetation structure was also influenced by lateral marsh retreat. For both *S. alterniflora*, which was dominant at 0 and 5 m from the edge, and high marsh species, which were dominant 15 m from the edge (i.e., on the ridge), VI was variable, but generally high, for rates of edge erosion of <2 m yr^−1^ and very low for lateral retreat rates of >8 m yr^−1^ (Figs S6 and S7). Despite the loss of vegetation close to the edge, distinct zones of species were maintained even where the retreat rates were so high (up to 14 m yr^−1^, Fig. 1) that the whole ridge was replaced within one year. Thus, high rates of marsh retreat did not cause a progressive loss of vegetation, but rather triggered an inland migration of the ridge and associated plant communities.

### Model of marsh morphodynamics and plant species interactions

**Figure 4:**
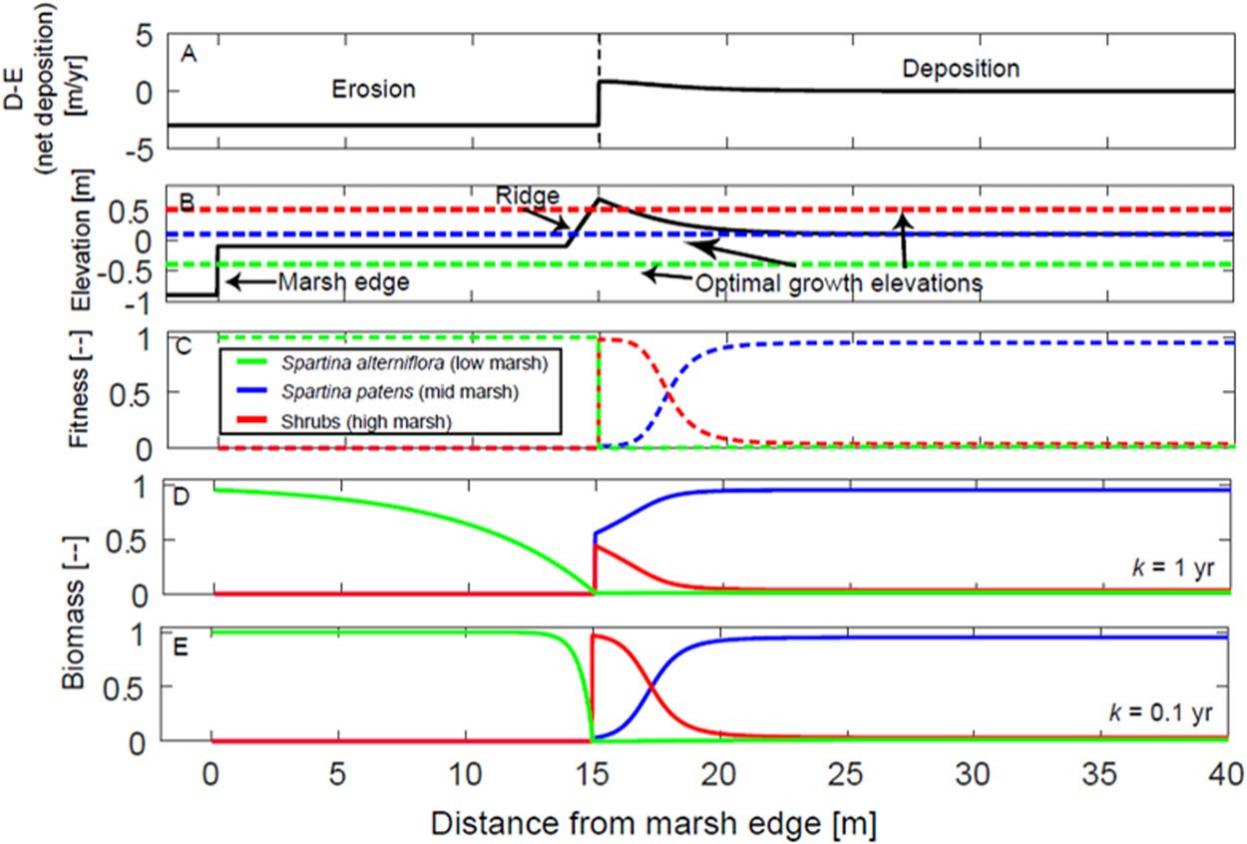
Model results at steady state under a marsh edge retreat rate of 5 m/yr. The position of the marsh edge is set at *x* = 0. (**A**) Net deposition rate; (**B**) Marsh elevation relative to MSL, showing the presence of a low marsh, a ridge, and a mid-elevation marsh. Elevation optima for the three different vegetation groups are shown; (**C**) Fitness functions; and (**D**,**E**) Vegetation abundance for two different values of the adaption time *k*. For a high value of *k* (**D**), the vegetation abundance deviates from the fitness function and the shrubs do not significantly develop on the ridge. For a low value of *k* (**E**) the vegetation abundance closely mirrors the fitness function and high marsh shrubs dominate the ridge.

To investigate the importance of gradients in physical stress and the disturbance of sedimentation events on plant species interactions, we developed a simple ecogeomorphic model. The model simulates a shore-normal transect across the marsh platform starting from the marsh edge, which migrates landward at a rate *M* (m yr^−1^). The evolution of the elevation *z* is described by a balance between wave erosion, *E*, and deposition, *D*, as $\dot{z}=D-E$ (Fig. 4A). The erosion term *E* is equal to a constant value up to the distance *L* from the marsh edge and then becomes zero, simulating the rapid decay of wave action away from the marsh edge. Because waves are only able to erode newly deposited sediment above the marsh platform but not the consolidated sediment, wave erosion only takes place up to the elevation *z*_*base*_, which is set equal to the low marsh elevation. The deposition term *D* is zero up to distance *L* and then increases to a value of *D*_*o*_, followed by an exponential decay at a rate *α. D*_*o*_ is set equal to the edge erosion rate, *M*, multiplied by a constant factor *β*, which describes the amount of material that is conserved and locally redeposited as the marsh retreats. Noticeably, the factor *β* should be close to zero in the presence of muddy sediment, which once eroded is quickly transported away, whereas it should be closer to one in the presence of sandy sediment, which redeposits close to the location it is eroded from.

Following a previous model for multi-species marsh plant competition as a function of elevation^23^, we introduce the elevation fitness function,${f}_{i}=2/(exp({\lambda }_{i}z/(z-z{o}_{i}))+exp(\,-\,{\lambda }_{i}z/(z-z{o}_{i})))$ where *zo*_*i*_ is the optimum elevation for the species *i*, and *λ*_*i*_ defines the spreading of the fitness function with elevation (Fig. 4B,C). The wave disturbance effect (causing plant shearing and breakage) is introduced by setting the vegetation fitness equal to zero in areas where wave erosion takes place (i.e., where *E* > 0) for stress-intolerant species, such as *S. patens* and high marsh shrub species^9^ (Fig. 4A).

The evolution of the normalized vegetation biomass, *X*_i_, stems from growth, set proportional to the relative fitness ${f}_{i}/[{\sum }_{j}{f}_{j}]$, and decay, set proportional to *X*_*i*_. The governing equation thus reads $\mathop{{X}_{i}}\limits^{\cdot }=1/k({f}_{i}/[{\sum }_{j}{f}_{j}]-{X}_{i})$, where *k* is the adaption time (yr), which is the time scale needed for plants to adapt to the relative fitness. Adaptation time can also be considered as the inverse of the normalized growth rate, which is the growth rate (biomass/time) divided by the asymptotic value (biomass at steady state). According to this simple model, when the adaption time is short, the vegetation quickly approaches steady state, which is characterized by *X*_*i*_ equal to ${f}_{i}/[{\sum }_{j}\,{f}_{j}]$. The limit case of *k* equal to zero is equivalent to the model of Marani *et al*. (2013): plants have no residual fitness from the previous year and their biomass – or the expected value of the biomass if a stochastic model is considered^23^ – adapts instantaneously to the present fitness conditions. If the adaption time is long, the vegetation has memory and its biomass reflects the biomass of the previous year. As a reference, the adaption time is set equal to one year, which means that a newly seeded plant (with no memory of the past season, e.g., without roots and rhizomes) reaches 63% of the steady state biomass within a year and 87% of the steady state biomass within two years.

To contextualize the model with the Delaware Bay system, the parameter *L* was set equal to the distance between the marsh edge and the ridge (~15 m, Fig. 1D), the parameter *β* was set equal to 0.5 to match observed deposition rates, the decay rate for the deposition was set equal to 0.5 m^−1^ to reproduce the observed ridge width based on LiDAR data. Edge erosion *M* was 1–20 m yr^−1^, depending on the model simulation, resulting in *D*_*o*_ values of 0.5 to 10 m yr^−1^. Edge erosion rates of 2 m yr^−1^, representative of historical to recent rates of edge erosion^27^, resulted in a *D*_*o*_ value of 1 m yr^−1^, which matches the maximum accretion rate measured at the ridge. *E* was set equal to 3 m yr^−1^ to match the seaward geometry of the ridge. Vegetation parameters were chosen to match field observations and mirror previous models^23^: optimal elevation for *S. alterniflora* was set to be between MSL and the low marsh elevation (0.6 m above MSL), optimal elevation for *S. patens* was set equal to the elevation of the mid-marsh platform (1.1 m above MSL), and the optimal elevation for shrubs was set to 1.5 m above MSL^11^ (Fig. S7). The spreading parameter, λ, was set to 10 (unitless).

## Model Results and Discussion

The model recreates a bulldozer-like effect, where the whole edge-platform-ridge profile migrates landward in a dynamic equilibrium, similar to a previous model for eolian dune migration^30^. The rapid transgression of marsh topography requires quick adaptation of the vegetation to preserve the floral zones as a cohesive unit ^e.g.,^^15^. In both the low marsh and the marsh plain, the vegetation matches the local fitness: the low marsh is dominated by *S. alterniflora* as waves remove stress-intolerant plants, whereas the marsh plain is dominated by *S. patens*, which has a competitive advantage over stress-tolerant species (Fig. 4D). Noticeably, the vegetation on the ridge deviates from the local fitness, i.e., *S. patens* dominates over the shrub species despite the latter having higher fitness at that elevation. This arises from the time limitation for shrubs to colonize the ridge when in competition with *S. patens* prior to experiencing the effect of edge retreat and thus succumbing to wave stresses (Fig. 4E). In order for the shrub community to dominate on the ridge, the adaption time needs to be shorter, e.g., to 0.1 yr, thus allowing rapid colonization and establishment (Fig. 4E).

We suggest that this shorter adaption time is provided by the wave disturbance. Specifically, we suggest that sediment deposition eliminates or reduces interspecific competition with dense perennial grasses, thus allowing new high marsh species to colonize the ridge more readily. Indeed, newly deposited sandy sediment provides a well-drained substrate which can be readily colonized by high marsh plants, while wrack smothers existing plants and traps additional sediments before colonization. Multiple processes including vegetative colonization, stochastic dispersal, seedling establishment, and competition must occur on time-scales shorter than ridge migration. In general, disturbances tend to favor colonization by seed^31^ and create increased resource availability (e.g., higher light levels) favorable to seed germination and seedling emergence for many species^32,33^. Species such as *P. australis*, an invasive grass, often found at the high marsh – upland transition, readily exploits these disturbances due to its prolific seed production^34^, widespread seed dispersal^35^, and persistence in the seed bank^36^. Similarly, the spread of *P. australis* both along the shoreline^37^ and in the high marsh^19^ has been attributed to sediment and/or wrack deposition that elevate the substrate above mean high water^38^. These model results agree with previous studies that have shown that sediment deposition events can stimulate low marsh grass productivity only up to an approximate threshold depth of 30 cm; above this threshold mortality occurs thus allowing the colonization of high marsh species via seed dispersal and seedling recruitment^39,40^.

Noticeably, the effect of wrack deposition on the ridge differed from what is commonly observed in the marsh interior. Wrack deposited in the marsh interior smothers extant vegetation and can trigger a loss of elevation and soil carbon^41^. Following eventual wrack decay, vegetation succession may begin with the initial colonization of salt tolerant species^8^ or, alternatively, ponds may form^42,43^. On the contrary, at the marsh edge, wrack was generally admixed with sand deposits resulting in an alternative ecological trajectory where the ridge substrate provided relatively porous, aerated conditions that promoted the rapid decay of wrack and colonization of high marsh species. Thus, the combined effect of sand and wrack deposition on the ridge was to accelerate colonization by fugitive and high marsh plants.

**Figure 5:**
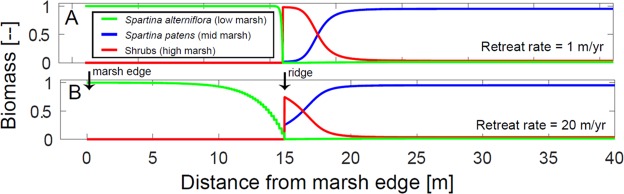
Comparison of the plant distribution for different marsh edge retreat rates (with the same adaption time equal to 0.1 years). (**A**) Retreat rate of 1 m/yr; and (**B**) Retreat rate of 20 m/yr. Note that in the former case the shrub vegetation on the ridge and the *S. alterniflora* in front of the ridge are higher than in the latter case.

Finally, even in the presence of disturbances (i.e., short adaption time), very fast rates of marsh retreat (20 m yr^−1^) resulted in lower shrub biomass on the ridge and lower *S. alterniflora* biomass in front of the ridge (Fig. 5). This result agrees with the field observations of low vegetation structure in areas with the fastest edge retreat rates (Fig. S6). This finding also supports the idea that in the presence of fast changing physical conditions, plant biomass (or the expected value of the biomass) is not necessarily in equilibrium with the local fitness as assumed in previous models^23^. For example, the model predicts that for extremely fast rates of marsh edge retreat (30 m yr^−1^), high marsh species would not be able to colonize the ridge.

Parameterizing the effect of disturbances by modifying the adaption time is a simple and efficient approach to simulate complex ecological interactions. This approach might complement the “windows of opportunity” approach, which recently has been successfully applied to simulate the encroachment of *Spartina* on the bare mudflat facing the marsh edge^44^. The adaption time approach can be useful in other ecotones with different types of competing plant species (e.g., marshes vs mangroves, or mangroves vs terrestrial vegetation) and different physical disturbances (e.g., episodic flooding, salinity, droughts, fires, and diseases).

Importantly, our observations and model results support existing theories explaining patterns of species richness and niche separation. Low species richness or outright mortality is predicted to correspond to a high frequency and intensity of disturbances, particularly where environmental stress is also high^45^, similar to conditions at the marsh edge. Wave disturbance weakens and abiotic stress declines with increasing distance from the marsh edge, increasing the potential for species interactions and increasing the number of possible species. In the absence of sediment deposition and ridge formation, which is predicted by the model in the case of *β* equal to zero (i.e., in the absence of sand re-deposition), low marsh species would directly transition to mid-marsh grasses as the result of competitive exclusion. Model results contribute to mounting support that disturbances increase the rate of competitive exclusion ^e.g.,^^46^, in this case, by allowing rapid colonization and competitive exclusion of ridge species over mid-marsh grasses. The maintenance of these vegetation zones, albeit in non-equilibrium with the local fitness as the marsh edge and ridge migrate landward, is a function of metacommunity dynamics where localized disturbance, dispersal ability, and rapid growth rates facilitate the near-continuous re-colonization of the retreating marsh edge and ridge. The ridge community is comprised of fugitive species, which are poor competitors but efficient colonizers, as well as high marsh species, which are competitively-dominant. Overall, the combination of frequent disturbances and a strong abiotic gradient (i.e., elevation) provides temporal niche opportunities for spatial differentiation among species creating a relatively diverse co-existence of species in an otherwise species-poor environment^47^.

## Conclusions

Waves are agents of recurrent catastrophic (vegetation removal) and non-catastrophic (vegetation stress) disturbances. In Delaware Bay, energetic waves and sand availability on the intertidal flat create sharp gradients in physical-sedimentary disturbances within 20 m of the marsh edge. These gradients structure the vegetation community into distinct zones that differ from the gradual elevation-driven zonation exhibited in the marsh interior. An abrupt shift in species composition occurs where bed stresses reduce wave energy and sediments fall out of the water column. There, stress-tolerant species shift to competitive high marsh species. The reduction in the plant adaption time associated with the deposition disturbances allow the high-marsh plant community to colonize the ridge during the relatively short time period between formation and destruction of the ridge.

Sea-level rise and marsh edge erosion have the potential to cause net marsh loss as well as replacement of high marsh habitat with low marsh habitat, especially where the marsh is subject to coastal squeeze and is impeded to transgress inland. This loss of high marsh habitat reduces the overall diversity and complexity of the marsh vegetation community, potentially leading to negative effects on wildlife species that depend on high marsh habitat (e.g.)^48^. In Delaware Bay, edge erosion is occurring more rapidly than landward transgression of the marsh into upland habitat^49^, indicating that high marsh species at the upland border may be lost. Here, we show that the presence of ridge near the marsh edge creates a novel high marsh habitat in an otherwise low marsh landscape. The disturbance associated with wave deposition allows rapid colonization of fugitive and high marsh species, allowing this community to transgress landward in dynamic equilibrium with the rapidly moving landscape. Thus, while the process of edge erosion is causing a net marsh loss, a high marsh habitat is created and maintained, providing a diverse and accessible habitat for wetland biota.

## Materials and Methods

The Delaware Estuary is a drowned valley of the Delaware River extending approximately 200 km from the mouth at Cape May, NJ to the head of tide at Trenton, NJ. The Estuary is characterized by semidiurnal tides with a tidal range that is amplified up-estuary from approximately 1.5 m at the mouth to approximately 3.0 m in Trenton, NJ. Sea-level rise rates averaged 3.5 mm per year over the last century due to a combination of eustatic sea-level rise and post-glacial subsidence^26^. While hard structures border much of the upper urban corridor from Wilmington, Delaware to Trenton, New Jersey, the middle and lower estuary is bordered primarily by tidal marshes and creeks interspersed with sandy beaches. Delaware Bay wetlands formed over unconsolodated Pleistocene sediments during Holocene sea level rise, and have since switched from constructive to destructive^50^. Wave activity at the edge of the tidal wetlands erodes the aggraded Holocene marsh and builds migrating sandy washover barriers^51^. Remote images and LiDAR show a characteristic sediment ridge on the marsh surface ringing much of the wetland-dominated lower estuary (Fig. 1). To characterize the location and distance of the sediment ridge from the marsh edge and determine relationships between ridge migration and marsh edge erosion, satellite images from 1991 and 2017 were used.

A field investigation was conducted to examine relationships between vegetation structure and composition and lateral erosion and sediment deposition along two >2 km stretches of marsh shoreline experiencing relatively high rates of long-term retreat (>5 m yr^−1^) on opposite sides of the Delaware Estuary. Bombay Hook (BH) on the western shore included the mouth of the Leipsic River in Bombay Hook National Wildlife Refuge and Stow Creek (SC) on the eastern shore included the mouth of Stow Creek. Stow Creek is near the estuarine turbidity maximum zone, which extends 70–120 km up-estuary where both suspended sediment concentrations and mudfloc sizes can be larger than areas up- and downstream^52^. The transition from tidal flat to marsh was variable alongshore, showing stepped profiles, erosional cliffs, undercutting, gentle and steep slopes, tidal gullies and islands.

At each study location, five transects perpendicular to the shoreline were established from approximately 10 m seaward to 15 m landward of the marsh edge. RTK surveys were repeatedly conducted along transects to examine changes in marsh edge profiles over a two-year period. Repeated GPS surveys of marsh profiles were conducted using a Leica GS 14 (Geoid 12 A coordinate system DE NAD83). Latitude, longitude and elevation data were collected approximately every 2.5 m along five transects from the intertidal flat to a 15 m distance into the marsh in July 2014, May and October 2015, and July and October 2016.

Lateral erosion rates at three depths on the marsh scarp were measured by use of erosion pins constructed of 2 m long rebar welded to square plates. Erosion pins were inserted horizontally at the live root zone (5 cm below the surface), below the root zone characterized by fine grained muds and well decomposed organic matter, and approximately 65–100 cm below the marsh surface and ~25 cm above the tidal flat (base). Measurements were made from the base of pin head to the marsh edge on each of the four sides over 827 days. In the event of erosion pin loss, RTK measurements of changes in marsh profile were used to estimate erosion. Erosion rates were calculated based on significant regressions of marsh edge movement for each pin.

Permanent vegetation and marker horizon plots were established at the marsh edge (0 m), and 5 and 15 m distances landward from the marsh edge. Plant species cover and height, and stem density were measured in 0.25 m^2^ permanent plots at 0, 5, and 15 m from the marsh edge along each transect. Measurements were made seven times from July 2014–2016. For analysis, a vegetation structure index (VI) was calculated as the product of species richness, stem density, and average canopy height^29^. This multiplicative calculation method was chosen due to its sensitivity to the number of species^53^. Because salt marshes are largely monospecific, we were interested in reflecting the weight of increasing species richness to the overall vegetation structure (vs. just simply measures of biomass − height × density). VI was calculated separately for the stress-tolerant species near the marsh edge, *S. alterniflora*, and the high marsh and fugitive species. Feldspar marker horizons were installed in July 2014 in 50 × 50 cm plots following the methodology of 28. Vertical accretion was measured as the change in height above the marker along three sides of a square plug cut from the plot. Accretion measurements were collected up to seven times from July 2014 to October 2016. Sediment deposition on the marsh surface was measured at 5 and 15 m from the edge by use of 15.5 cm^2^ aluminum sediment plates welded to anchor poles inserted vertically into the marsh. Care was taken to adjust the surface of plates flush with the marsh surface. Sediment deposited on the plates was collected seven times from July 2014 to October 2016, at two to four month intervals.

### Data analysis

Linear regression analysis were used to test the relationships between distance from the marsh edge, elevation, sediment deposition rate, and vegetation indices. Erosion rate data were natural log-transformed prior to statistical analysis. The influence of erosion, elevation, and sediment deposition rates on VI of were tested using regression analysis.

## Supplementary information

Supplementary Information

## Data Availability

All data generated and analyzed during this study are included in this published article (and its Supplementary Information files).
