# Supplementary material for: Retreating marsh shoreline creates hotspots of high-marsh plant diversity
Source: Sci Rep. 2019 Apr 8;9:5795. doi: 10.1038/s41598-019-42119-8 (PMC6453922; doi:10.1038/s41598-019-42119-8)
Supplement: Supplementary file 1 — Supplementary Information [file 41598_2019_42119_MOESM1_ESM.docx]

**Supplementary Information**

**Retreating marsh shoreline creates hotspots of high-marsh plant diversity**

Tracy Elsey-Quirk

Giulio Mariotti

Kendall Valentine

Kirk Raper

SUPPLMENTAL TABLES AND FIGURES

Table S1. Elevation and lateral erosion rate of study locations.

| Site | Transect | Elevation  (m, NAVD88) | Root zone lateral  erosion rate  (m yr^-1^) |
| --- | --- | --- | --- |
| BH | T1 | 0.979 | 1.07 ± 0.14 |
|  | T2 | 0.600 | 9.45 ± 1.42* |
|  | T3 | 0.672 | 0.91± 0.10 |
|  | T4 | -0.164 | 1.00 ± 0.54 |
|  | T5 | 0.733 | 11.19 ± 1.46* |
| SC | T1 | 0.922 | 0.12 ± 0.01 |
|  | T2 | 0.449 | 2.50 ± 0.23 |
|  | T3 | 0.687 | 0.12 ± 0.02 |
|  | T4 | 0.703 | 0.09 ± 0.01 |
|  | T5 | 0.297 | 2.27 ± 0.13 |
| *erosion pin lost; estimated from RTK survey | | | |

**

**

Fig. S1. Average canopy height of species (upper) and sedimentation and surface accretion rate determined from sediment plates and marker horizons, respectively (lower) in permanent plots originally placed at 0, 5 and 15 m inland from the marsh edge. All non-vine plant species above a 10 cm height were included. Note: permanent plots became increasing closer to the marsh edge over the two year study period (represented in Fig. 3).


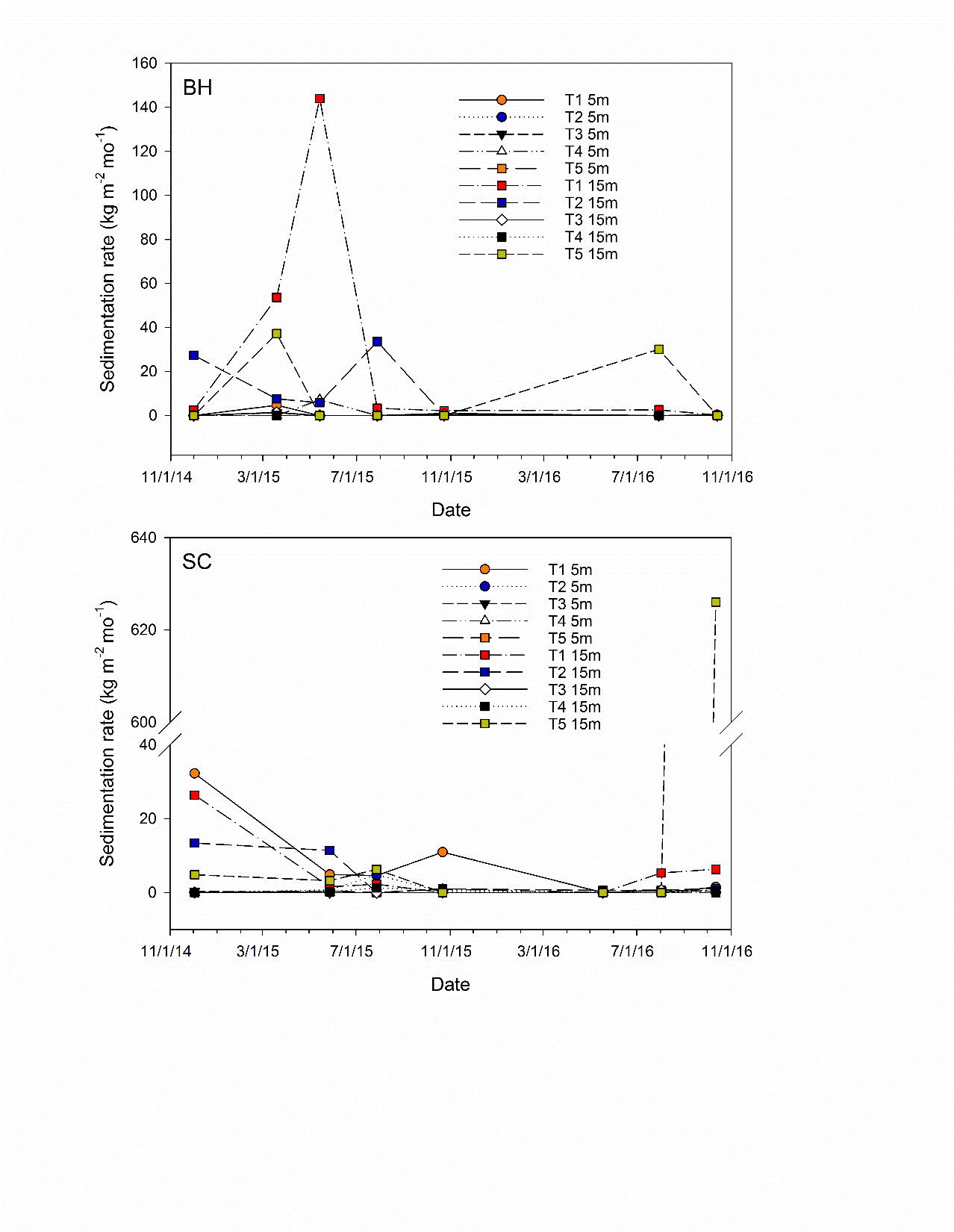


**60% OM**

**8% OM**

**68% OM**

**3% OM**

**2% OM**

Fig. S2. Sedimentation rate at 5 and 15 m from the edge along 5 transects at BH and SC over time. Percent organic matter is shown for a subset of values. Note differences in y-axis scale and distance designations changed over time as the marsh eroded (represented in Fig. 3)


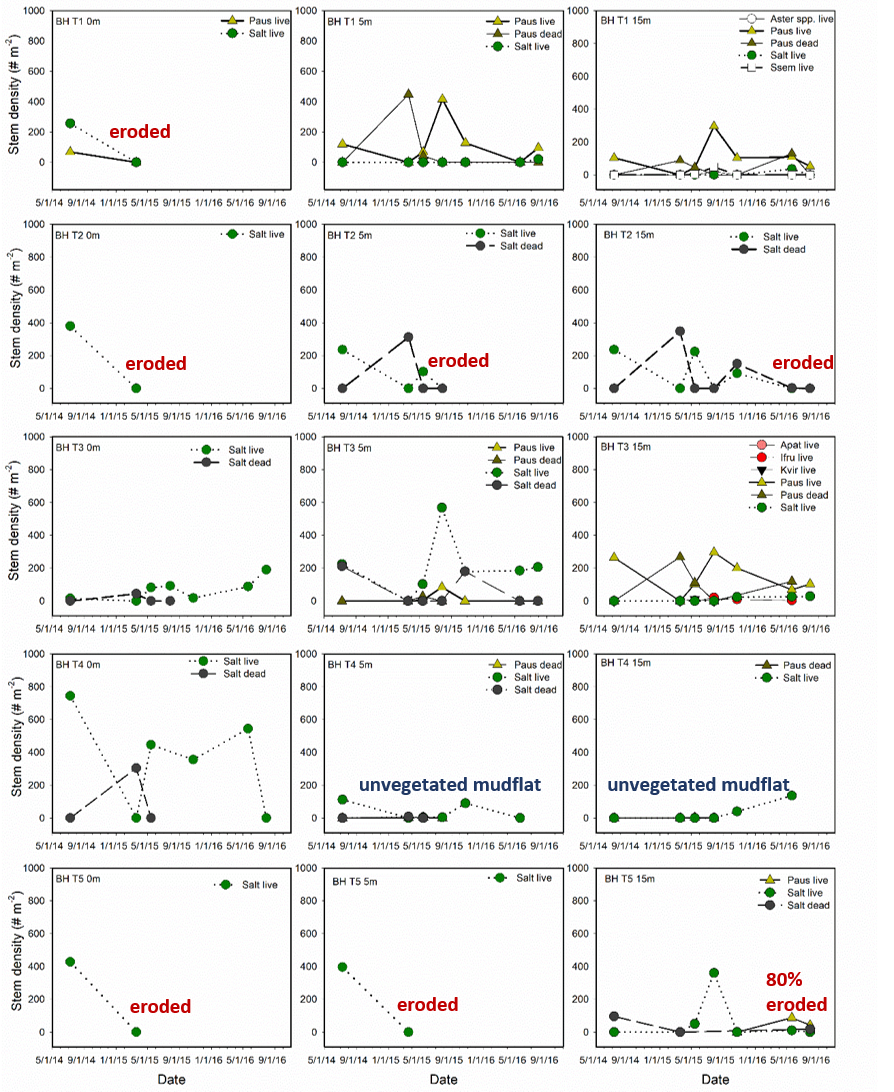


Fig. S3. Time series of species stem density at the marsh edge (0 m), 5 and 15 m landward in BH, Delaware Estuary. Note distance designations changed over time as the marsh eroded (represented in Fig. 3). Species abbreviations: *Atriplex patula* (Apat); *Iva frutescens* (Ifru); *Kostelytzkya virginica* (Kvir); *Phragmites australis* (Paus); *Spartina alterniflora* (Salt).


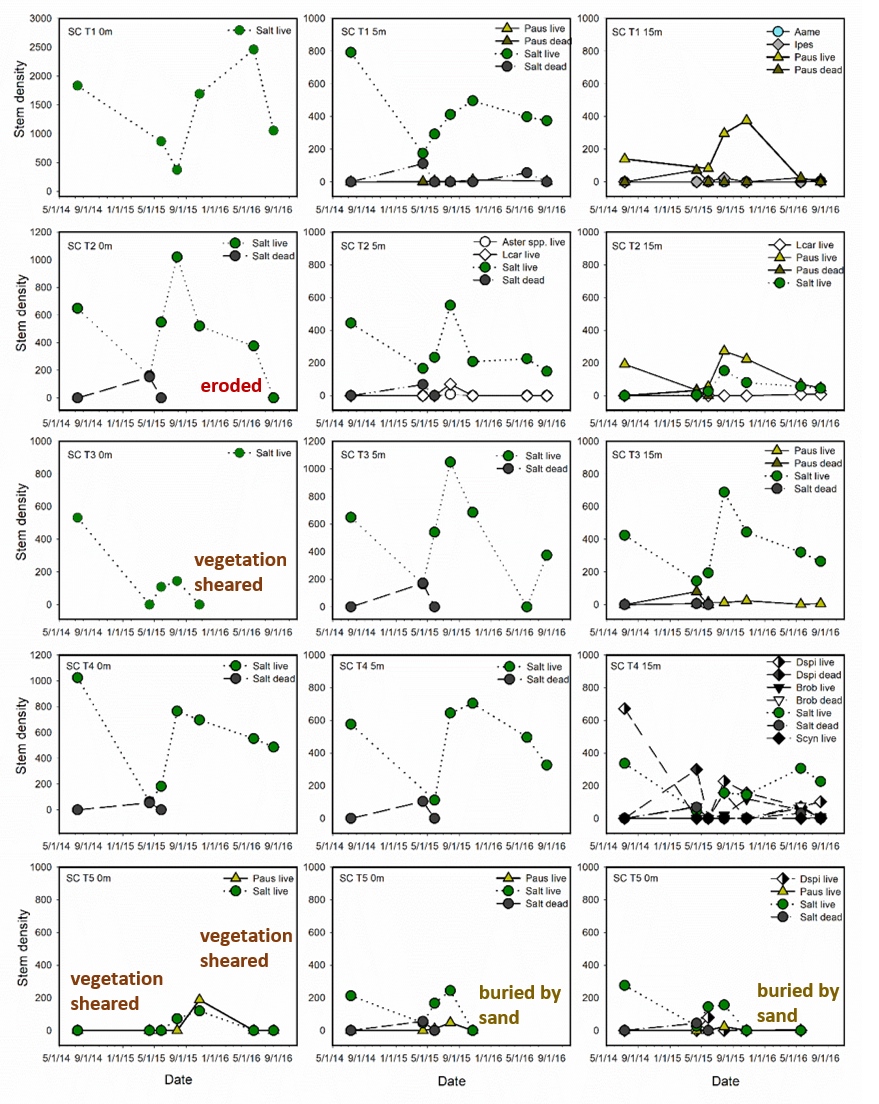


Fig. S4. Time series of species stem density at the marsh edge (0 m), 5 and 15 m landward in SC, Delaware Estuary. Note differences in y-axis scale and distance designations changed over time as the marsh eroded (represented in Fig. 3). Species abbreviations: *Apios Americana* (Aame); *Bolboshoenus robustus* (Brob); *Spartina cynosyroides* (Scyn); *Distichlis spicata* (Dspi); *Ipomea pes-caprae* (Ipes); *Limonium caroliniana* (Lcar).


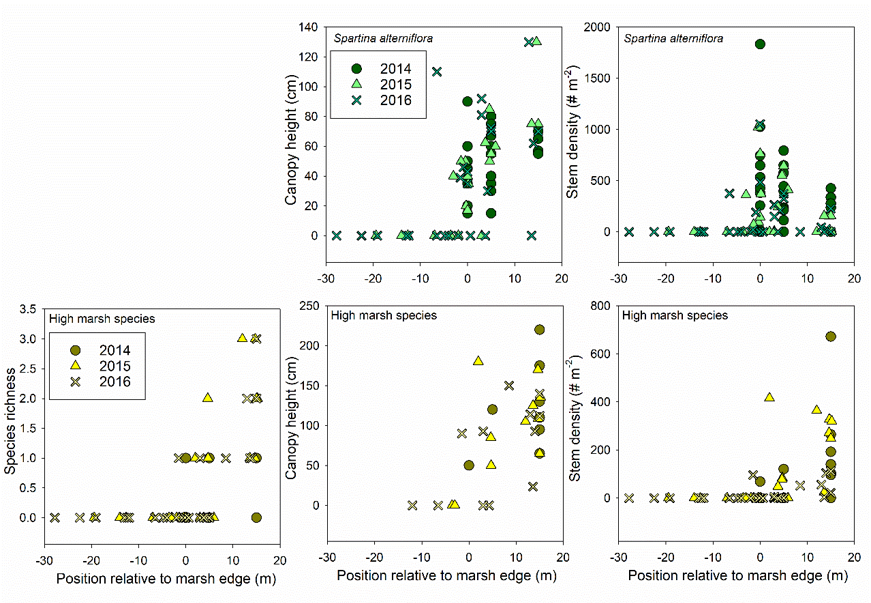


Fig. S5. Vegetation structural components (i.e., species richness, canopy height, and stem density) of the low marsh stress-tolerant species, *S. alterniflora* (green) and the high marsh plant community (yellow) at the estuary-marsh transition over a three year period.





Fig. S6. Relationship between the vegetation structure index (VI) and annual retreat rate for *S. alterniflora* at 0 and 5 m and high marsh species at 15 m distance from the marsh edge. Natural log-transformed erosion rate data are displayed.





Fig. S7. Canopy height and stem density of *S. alterniflora* (top) and the high marsh plant community (bottom) at the estuary-marsh transition at different rates of edge erosion.


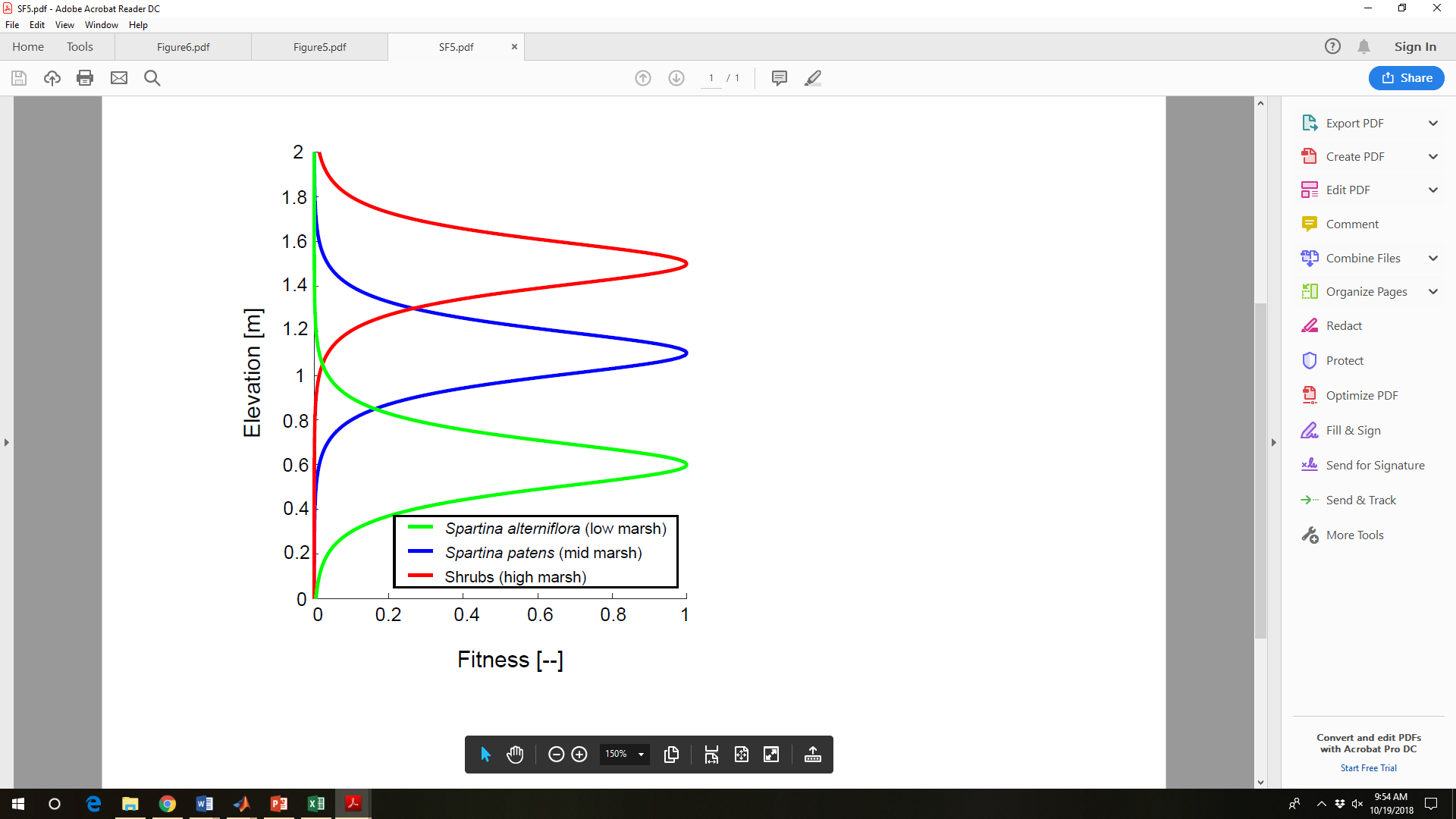


Fig. S8. Fitness of a given plant class per elevation.
